# Electrical Detection of the Helical Spin Texture in a p-type Topological Insulator Sb_2_Te_3_

**DOI:** 10.1038/srep29533

**Published:** 2016-07-11

**Authors:** C. H. Li, O. M. J. van ‘t Erve, Y. Y. Li, L. Li, B. T. Jonker

**Affiliations:** 1 Materials Science and Technology Division, Naval Research Laboratory, Washington, DC 20375, United States; 2 Department of Physics, University of Wisconsin, Milwaukee, WI 53211, United States

## Abstract

The surface states of 3D topological insulators (TIs) exhibit a helical spin texture with spin locked at right angles with momentum. The chirality of this spin texture is expected to invert crossing the Dirac point, a property that has been experimentally observed by optical probes. Here, we directly determine the chirality below the Dirac point by electrically detecting spin-momentum locking in surface states of a p-type TI, Sb_2_Te_3._ A current flowing in the Sb_2_Te_3_ surface states generates a net spin polarization due to spin-momentum locking, which is electrically detected as a voltage on an Fe/Al_2_O_3_ tunnel barrier detector. Measurements of this voltage as a function of current direction and detector magnetization indicate that hole spin-momentum locking follows the right-hand rule, opposite that of electron, providing direct confirmation that the chirality is indeed inverted below Dirac point. The spin signal is linear with current, and exhibits a temperature dependence consistent with the semiconducting nature of the TI film and freeze-out of bulk conduction below 100 K. Our results demonstrate that the chirality of the helical spin texture of TI surface states can be determined electrically, an enabling step in the electrical manipulation of spins in next generation TI-based quantum devices.

Three-dimensional (3D) topological insulators (TIs) form a new quantum phase of matter in which metallic surface states populated by Dirac fermions coexist with an insulating bulk^1^,^2^,^3^,^4^,^5^. These states are topologically protected by time reversal symmetry and exhibit intriguing properties for spintronic applications^6^,^7^,^8^,^9^,^10^. One of the most striking properties is that *of spin-momentum locking*–the spin of the Dirac fermion is predicted to be locked perpendicular to its momentum^11^,^12^,^13^, as illustrated in Fig. 1a. This property was first confirmed by surface sensitive spin- and angle-resolved photoemission spectroscopy (ARPES)^8^,^9^,^10^ and polarized optical spectroscopic techniques^14^. Our recent work showed that when a small bias current flows in the surface states of the *n*-type TI Bi_2_Se_3_, the spin-momentum locking generates a net spin polarization that can be *electrically* detected as a voltage on a ferromagnetic tunnel barrier contact, as shown schematically in Fig. 1b 15, where the voltage is proportional to the projection of the spin polarization onto the magnetization. Similar results were subsequently obtained in related ternary and quaternary TI alloys^16^,^17^,^18^,^19^,^20^. Furthermore, the spin-momentum has also shown to exert a strong spin-transfer torque in a Bi_2_Se_3_/ferromagnetic heterostructure at room temperature^21^, and a three orders of magnitude enhancement in in-plane charge current induced magnetic switch efficiency in (Bi_0.5_Sb_0.5_)_2_Te_3_/(Cr_0.08_Bi_0.54_Sb_0.38_)_2_Te_3_ reported for conventional method^22^,^23^.

Interestingly, the helical nature of TI surface states, as manifested in the spin momentum locking, also exhibits a chirality that inverts above and below the Dirac point, E_D_, so that the carrier spin and momentum locking follows a left-hand rule when the Fermi level (E_F_) is above the Dirac point, and right-hand rule below^24^,^25^,^26^ (Fig. 1a). Above the Dirac point, surface-state spins are tangential to the Fermi surface contour with clockwise helicity, i.e., a quasi-particle (electron) moving in the +k direction is locked with −y spin polarization state, whereas below the Dirac point, the +k moving quasi-particle (hole) is locked with +y spin and exhibits counterclockwise helicity. Again, this chirality inversion has been explicitly observed experimentally by spin-resolved ARPES in BiTlSe_2_ and Bi_2_Se_3_^24^,^25^. However, direct electrical access to this varying spin texture of the TI surface states has not been realized in a p-type 3D TI.

Here we demonstrate the determination of the chirality of the spin texture below the Dirac point using transport measurement, through the direct electrical detection of spin-momentum locking in a p-type TI, Sb_2_Te_3_, probing the spin texture of the TI surface states below the Dirac point. We use Fe/Al_2_O_3_ tunnel barrier contacts to detect bias current generated spin polarization. From measurements of the carrier type and sign of the spin voltage, we deduce a counter-clockwise chirality with carrier spin-momentum locking following a right-hand rule, confirming the chirality inversion of the helical spin texture of TI surface state above and below the Dirac point. The direct electrical access to the helical spin texture in the TI surface states is an enabling step in the electrical manipulation of spins in topological devices for spintronics and quantum computation applications.

## Results

### MBE growth and characterization of Sb_2_Te_3_

**Figure 2:**
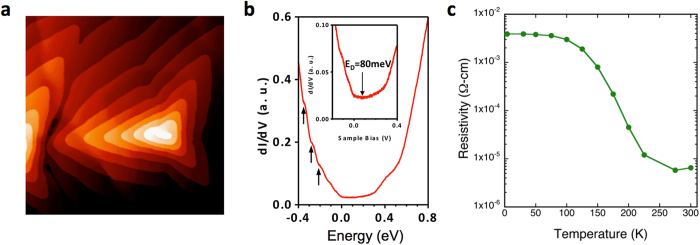
Structural and electrical characterization of the MBE Sb_2_Te_3_ films. (**a**) STM image of the MBE grown Sb_2_Te_3_ film (500 × 500 nm^2^, I_t_ = 0.1 nA, V_s_ = −1.0 V). (**b**) Tunneling spectra taken at 77 K, where the minimum conduction point at ~80 meV above the Fermi level is attributed to the Dirac point, indicating p-type conductivity. Arrows point to quantum well states observed. (**c**) Temperature dependent resistivity of the TI film showing over two orders of magnitude decrease in resistance from 300 to 15 K, indicating semiconducting behavior.

Sb_2_Te_3_ thin films 30 nm thick are grown by molecular beam epitaxy (MBE) on epitaxial graphene/SiC(0001) substrates at 275–325 °C (see Methods). A conductive nitrogen doped *n*-type 4H-SiC substrate (0.05 Ohms-cm) is used to facilitate the *in situ* scanning tunneling microscopy/spectroscopy (STM/STS) monitoring of the surface morphology and electronic structure to ensure optimal layer-by-layer spiral growth (Fig. 2a)^27^. The *n*-type substrate does not contribute to transport through the *p-*type TI film due to the depletion region at the *p-n* junction interface, as discussed in the Supplementary Information. Tunneling spectrum shown in Fig. 2b taken on the as-grown Sb_2_Te_3_ surface *in situ* at 77 K exhibits a minimum conductance at +80 meV above the Fermi level, indicating the Dirac point (E_D_) (identified by an arrow in the inset). This indicates p-type carriers in the TI surface states, with an estimated hole concentration of ~4.7 × 10^11^/cm^2^. The p-type conductivity is as also confirmed by *ex situ* Hall measurements, as shown in the Supplementary Information. This value is slightly enhanced due to the shift of the Dirac point to accommodate charges induced by the electric field between the STM tip and sample, as observed in earlier STS studies of Sb_2_Te_3_^28^. In addition, quantum well (QW) states (marked by arrows in Fig. 2b) are also observed, consistent with earlier STS^28^,^29^ and ARPES studies^30^.

Temperature dependent resistivity measurements show over two orders of magnitude increase in resistivity with decreasing temperature (Fig. 2c), indicating semiconducting behavior, *i.e.,* the Fermi level lies within the bandgap. A weakly temperature dependent plateau is seen below 100 K, characteristic of metallic behavior, and attributed to freeze-out of bulk carriers and conduction only in metallic states such as the TI surface states. These findings are consistent with earlier work on MBE grown Sb_2_Te_3_ films^28^,^29^,^30^, where *p*-type conductivity is typically found and attributed to excess Sb leading to Sb_Te_ acceptor-like antisites with an activation energy of ~7 meV^31^, where the Fermi level lies in the gap and intersecting only the surface Dirac cone^30^. This is in contrast to bulk Sb_2_Te_3_ single crystals synthesized by melting mixtures of Sb and Te, where much larger *p*-type doping is typically found with the Fermi level lying within the bulk valence band^32^.

### Device fabrication and measurement geometry

Ferromagnet/oxide tunnel contacts, Fe/Al_2_O_3_, are utilized as spin sensitive probes to detect the bias current-generated spin polarization in the Sb_2_Te_3_ surface states. Such contacts have enabled electrical detection of spin current and accumulation in both semiconductors and metals^33^ and recently spin-momentum locking in topological insulators such as Bi_2_Se_3_^15^,^16^,^18^,^19^,^20^.

**Figure 3:**
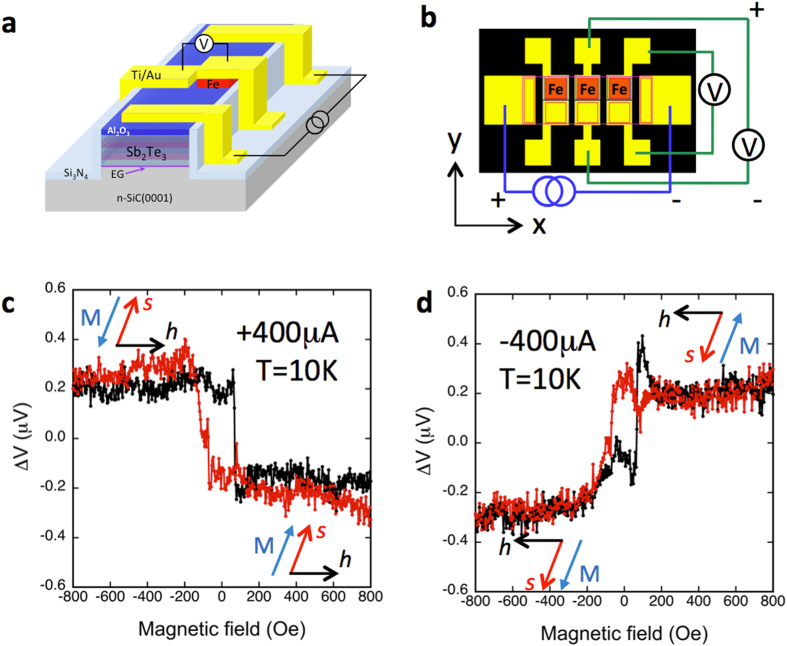
Schematic of the contacts and transport devices and TI spin polarization detected as a voltage with Fe/Al_2_O_3_ contacts. (**a**) Schematic and (**b**) top view of contact layout with two parallel rows of collinear detector contacts, top row is ferromagnetic (Fe, red), bottom row is non-magnetic reference (Ti/Au). The ferromagnetic contacts are 80 × 80 μm separated by 45 μm edge to edge, which are separated by 15 μm from the corresponding non-magnetic reference contacts. (**c**) Magnetic field dependence of the voltage measured at the ferromagnetic detector contact with the magnetization collinear with the induced TI spin for bias currents of +400 μA and (**d**) −400 μA.

MBE grown Sb_2_Te_3_ films were processed into the device structures illustrated in Fig. 3a,b for transport measurements, consisting of two Au/Ti current leads on opposite ends of the Sb_2_Te_3_ mesa, with several pairs of ferromagnetic (red) detector and corresponding non-magnetic Au/Ti (yellow) reference contacts in between. The unpolarized current flowing between the two outer contacts produces a spontaneous spin polarization in the Sb_2_Te_3_ surface states throughout the channel due to spin-momentum locking. The projection of this spin onto the magnetization of the ferromagnetic detector contact is recorded as a voltage with a high-impedance voltmeter (>1 Giga-ohm). Note that no current flows through the detector contact. An in-plane magnetic field is applied to switch the magnetization of the detector contact, so that the spins generated by carriers populating the Sb_2_Te_3_ surface states are either parallel or antiparallel to the magnetization, which changes the magnitude of detector voltage. Here we define the positive current to be holes flowing from left to right along the +*x* axis, and the positive magnetic field to be pointing in the +*y* direction.

### Electrical detection of spin-momentum locking

Transport measurements were carried out in a closed cycle cryostat equipped with an electromagnet (10–300 K, ±1000 Oe). Figure 3c,d show the detector voltage as a function of the applied in-plane magnetic field when a bias current flows between the two outer Ti/Au contacts. A simple linear background was subtracted^15^, and the data centered on the vertical axis. For a constant +400 uA current (Fig. 3c), a constant low voltage is observed when the detector magnetization is saturated in the +y direction (>70 Oe positive magnetic field), and a constant high voltage is observed with the detector magnetization in the −y direction (negative field). From our earlier work on the electrical detection of spin-momentum locking in Bi_2_Se_3_ using the same detector contacts^15^, when the detector magnetization is parallel to the TI spin, a low voltage is detected. (A complete description of the measurement and model is contained in ref. 34.). Given the same spin detecting contacts (Fe/Al_2_O_3_), which are only sensitive to the orientation of the spin regardless of the source, this indicates that in Fig. 3c the low voltage observed correspond to a spin orientation in the +y direction, locked to a hole momentum in the +x direction. This counter-clockwise helicity deduced here below the Dirac point in a p-type TI is indeed opposite to that above the Dirac point as seen in a n-type TI, where the +x moving electron induces a spin locked in the −y direction. This is further confirmed when the detector magnetization is saturated in the −y direction (negative field), where a high voltage is observed, indicating an antiparallel alignment between the detector magnetization and spin orientation, i.e., a spin orientation in the +y direction.

Another hallmark of the current generated spin in the TI surface states due to spin-momentum locking is that the spin orientation can be changed by reversing the current direction, as shown in Fig. 3d for −400 uA current, i.e., holes flowing in the −x direction. Following the discussion above, this should induce a spin direction in −y. A parallel alignment between this TI surface spin and that of detector magnetization (<−70 Oe, negative magnetic field) again results in a low voltage, while the antiparallel alignment (>+70 Oe, positive field) yields a high voltage. The resulting curve is a step-like hysteric field dependence of the FM detector voltage that flips around the zero voltage axis relative to that in Fig. 3c. The voltage measured with a non-magnetic detector exhibits no such step-like behavior. These results clearly show that the current generated spin orientation in the Sb_2_Te_3_ surface states is locked in a counterclockwise helicity to the hole momentum, demonstrating that chirality in spin-momentum locking is indeed inverted below the Dirac point.

### Bias & temperature dependence of spin signal

**Figure 4:**
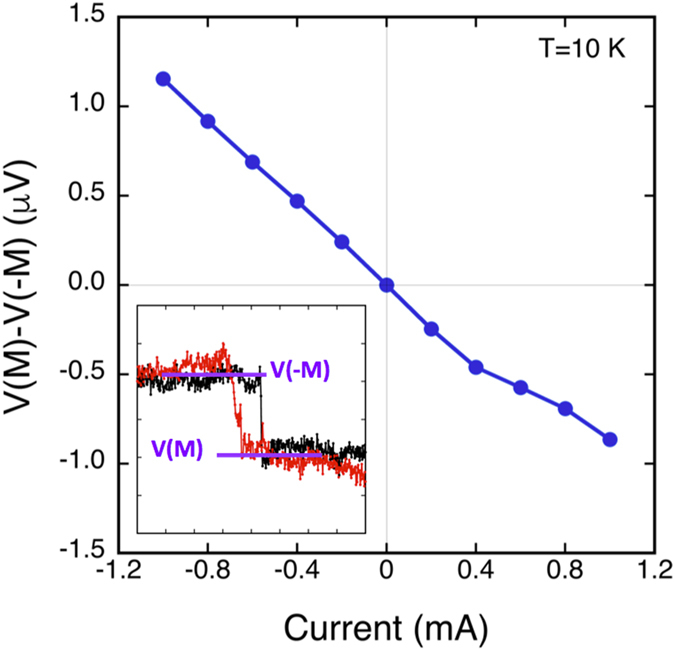
Bias current dependence of the ferromagnetic detector voltage. Height of the voltage hysteresis curve *ΔV* = *V*(*M*) *−* *V*(*−M*) above detector saturation field as a function of bias current. Inset: illustration of how *ΔV* is determined.

The change in detector voltage, *ΔV = V*(***M***) −* V*(*−**M***), produced when the detector magnetization reverses (the spin of the Sb_2_Te_3_ surface states is parallel or antiparallel to the detector magnetization) depends linearly upon the applied bias current that flows in the Sb_2_Te_3_ film, as shown in Fig. 4. *Δ*V is determined from the data as illustrated in the inset. This behavior is consistent with a spin polarization generated by a bias current, and also consistent with earlier theoretical work^35^ as discussed in more detail below.

**Figure 5:**
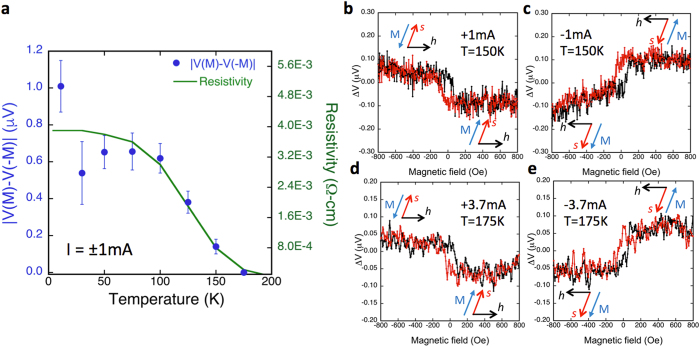
Temperature dependence of the spin voltage. (**a**) The height of the voltage hysteresis curve |*ΔV*| as a function of temperature for 1 mA bias current (blue dots), and temperature dependence of the resistivity of the TI film (green line). (**b**) The detector voltage as a function of magnetic field at 150 K at +1 mA and (**c**) −1 mA. (**d**) The detector voltage as a function of magnetic field at 175 K at +3.7 mA and (**e**) −3.7 mA.

The temperature dependence of the spin signal [*V*(***M***) −* V*(*−**M***)] measured at ±1 mA is shown in Fig. 5a. It initially exhibits a plateau as the temperature decreases below 100 K, where the transport measurements shown in Fig. 2c indicate the freeze out of some of the bulk carriers, and metallic states such as the TI surface states dominate transport. At higher temperatures, a significant decrease in magnitude is observed, and the signal disappears at this bias current above 175 K. The data taken at 150 K are shown in Fig. 5b,c, where a clear step-like hysteric behavior is seen. This temperature dependence is consistent with the semiconducting nature of the TI film, mirroring the temperature dependence of the resistivity (green curve in Fig. 5a). Since the conduction of the metallic states is expected to be weakly temperature dependent^36^, the significant decrease in resistance above 100 K is attributed to activation of bulk carriers such as Sb_Te_ antisites in the semiconducting TI bulk, which shunts increasing fractions of the current, and dilutes the portion that flows through the surface states. By increasing the total bias current from ±1 mA to ±3.7 mA at 175 K, a clear hysteretic step-like behavior is again seen (Fig. 5d,e).

## Discussion

The linear behavior of the spin signal is consistent with the theoretical work by Hong *et al*. on current-induced spin polarization in a TI in both diffusive and ballistic regimes^35^. The voltages measured on the FM detector *V*(***M***) are directly related to the current and spin polarization by [*V*(***M***) −* V*(*−**M***)] = *I*_*b*_ *R*_*B*_ *P*_*FM*_ (***p**. **M***_***u***_), (bold case denotes a vector) where *I*_*b*_ is the (hole) bias current in the +*x* direction, *R*_*B*_ is the ballistic resistance of the channel, and *P*_*FM*_ is the transport spin polarization of the FM detector metal. ***M***_***u***_ is a unit vector along the detector magnetization ***M***, and ***p*** is the degree of spin polarization induced per unit current by both spin-momentum locking in TI Dirac surface states and Rashba spin-orbit coupling in the two-dimensional electron or hole gas that may form on the surface due to band bending.

Though it has been shown that the surface of Sb_2_Te_3_ is much more stable against adsorbate-induced band bending and shifting of the Fermi level^30^, and exhibits less aging or photo-induced doping typical of Bi-based materials such as Bi_2_Se_3_^32^,^37^, the formation of a two dimensional hole (or electron) gas on the surface post processing cannot be ruled out. Due to the breaking of inversion symmetry at the Sb_2_Te_3_ surface, these states can exhibit Rashba-type spin-orbit splitting with spins in-plane and also at approximately right angles to the carrier momentum, and therefore may also contribute to the spin signal measured. However, these states exist as spin-split pairs with opposite spin orientation at each momentum, therefore the resulting current-induced spin densities tend to cancel. Hence the net spin polarization should be dominated by that from the TI surface state, as predicted by model calculations^35^. Furthermore, the TI surface state and Rashba contributions can also be distinguished since they are expected to exhibit opposite sign. Following Eqn. 1 and models presented in ref. 34, the sign of the spin voltage signal [*V*(***M***) −* V*(*−**M***)] that we measure (Fig. 3c,d) is consistent with contribution from the TI surface states. Finally, the sign of the spin signal/polarization measured here for a p-type TI is the same as we’ve shown previously for the n-type Bi_2_Se_3_^15^. This is also consistent with the prediction by Hong *et al*. (c.f. Fig. 3c of ref. 35), where the polarization of the TI surface states is shown to be constant with energy, and work of ref. 19 where the chemical potential of (Bi,Sb)_2_Te_3_ was tuned by a back gate^19^.

While a quantitative experimental determination of the TI spin polarization ***p*** can be obtained from the equation above, it is limited by the precise determination of the fraction of the current that flows through the TI surface states (which generates the spin polarization due to spin-momentum locking). Even though temperature dependent resistivity measurements suggest freeze out of bulk carriers at lower temperatures (<100 K, Fig. 2c), there may still be other conducting channels that contribute to transport and shunt the bias current. This is evident from the large disparity in carrier concentration measured in tunneling spectroscopy measurements carried out in ultrahigh vacuum that are most sensitive to surface (states) and Hall measurements which integrate over a range of energies. The Dirac point of +80 meV in the STS spectrum (Fig. 2b, taken at 77 K) indicates an Sb_2_Te_3_ surface state carrier density of 4.7 × 10^11^/cm^2^ in the as-grown sample, while Hall measurements at the same temperature yield a sheet density of 8.0 × 10^13^/cm^2^ in the processed sample corresponding to the data of Fig. 2c. Although adsorbates are known to introduce additional doping into the TI system on the order of ~10^12^/cm^2^ in the case of Bi_2_Se_3_, Sb_2_Te_3_ has been shown to be much less susceptible to such adsorbates induced effects^30^, and therefore is likely not the culprit for such drastic difference in sheet density.

Alternatively, earlier work has shown robust and well-defined QW states due to confinement in MBE grown Sb_2_Te_3_ thin films^29^,^30^, which is also clearly seen in our tunneling spectroscopy data (Fig. 2b) with a peak-to-peak separation of ~80 meV. These states are two-dimensional in nature and can exhibit a weak temperature dependence. However, these states can contribute to the sheet density measured by Hall measurements, and certainly contribute to transport and provide a parallel conduction path. As a first order approximation, we assume equal conduction through the bulk and the surface states, and that only the current flowing through the top surface contributes to the spin polarization arising from the Dirac states. With this assumption, and taking *P*_*FM*_(Fe) ~ 0.4, and *k*_*F*_ ~ 0.07 Å^−1 ^38 we estimate |***p***| ~ 0.15 from the data in Fig. 4.

In summary, we demonstrate that the chirality of the helical spin texture of a topological insulator can be determined using transport measurement, by electrically detecting the spin-momentum locking of the TI surface states. In the p-type TI, Sb_2_Te_3_, where the carrier type is confirmed by Hall measurement, using Fe/Al_2_O_3_ tunnel barrier contacts to detect bias current generated spin polarization, our results indicate a counter-clockwise chirality with carrier spin-momentum locking following a right-hand rule, confirming that the chirality is indeed inverted below the Dirac point. The spin signal arising from spin-momentum locking of the surface states is linear with current, and exhibits a temperature dependence consistent with the semiconducting nature of the TI film. With the efficient spin-momentum driven spin transfer torque^21^ and magnetization switching^22^ already demonstrated in TIs such as Bi_2_Se_3_, the direct electrical access to the spin texture of the helical TI surface states is an enabling step towards realizing next generation TI-based spintronics devices.

## Methods

The growth of Sb_2_Te_3_ films was carried out on epitaxial graphene/SiC(0001) substrates in an ultrahigh vacuum (UHV) system (base pressure ~1 × 10^−10^ Torr) that integrates two MBE chambers and a low temperature (5–300 K) scanning tunneling microscope (STM). As-received nitrogen-doped 4H-SiC(0001) substrates were first etched in H_2_/Ar atmosphere in a separate vacuum chamber at ~1500 °C to remove polishing damage. They were then transferred to the MBE system and annealed in UHV in a Si flux (~0.1 ML/min) at 950 °C to produce a (3 × 3) reconstructed surface, and further annealed at temperatures 1000–1300 °C without Si flux to grow epitaxial graphene^39^. For the growth of Sb_2_Te_3_, the substrate was held at 275–325 °C, and Sb and Te were supplied via separate Knudsen cells at 460 and 250 °C, respectively. *In situ* STM imaging is used to monitor surface morphology and electronic structure and ensure optimal layer-by-layer spiral growth, as shown in Fig. 2a. The as-grown film exhibits a Dirac point 80 meV above the Fermi level, as shown in the tunneling spectra in Fig. 2b, indicating p-type conductivity with an estimated carrier concentration of ~4.7 × 10^11^/cm^2^. These values represent an upper bound due to the upward shift of the Dirac point accommodating charges induced by the electric field between the STM tip and sample^28^.

The Fe/Al_2_O_3_ contacts were formed on the air-exposed surface of a 20 nm thick Sb_2_Te_3_ film in a separate MBE system as follows. A 0.7 nm layer of polycrystalline Al was first deposited by MBE, and then oxidized in 200 Torr O_2_ for 20 min in the presence of UV light in the load-lock chamber. This step was then repeated for a total Al_2_O_3_ thickness of 2 nm. The sample was then transferred under UHV to an interconnected metals MBE chamber, where 20 nm of polycrystalline Fe was deposited at room temperature from a Knudsen cell.

The samples were processed into the device structures illustrated in Fig. 3a,b to enable transport measurements. Standard photolithography and chemical etching methods were used to define the Fe contacts, which ranged in size from 20 × 20 μm^2^ to 80 × 80 μm^2^, with adjacent contact separation ranging from 45 to 200 μm. Ion milling was used to pattern the Sb_2_Te_3_ mesa. Large Ti/Au contacts were deposited by lift-off in an electron beam evaporator as non-magnetic reference contacts and bias current leads. The Fe contacts were capped with 10 nm Ti/100 nm Au, and bond pads for wire bonded electrical connections are electrically isolated from the SiC using 100 nm of Si_3_N_4_.

Transport measurements were performed in a closed cycle cryostat equipped with an electromagnet (4–300 K, ±1000 Oe). An unpolarized bias current was applied through the outer Ti/Au contacts on the opposite ends of the Sb_2_Te_3_ mesa, and the voltage on the detector contact was recorded as a function of the in-plane magnetic field applied orthogonal to the electron bias current direction in the TI.

## Additional Information

**How to cite this article**: Li, C. H. *et al*. Electrical Detection of the Helical Spin Texture in a p-type Topological Insulator Sb_2_Te_3_. *Sci. Rep.* **6**, 29533; doi: 10.1038/srep29533 (2016).

## Supplementary Material

Supplementary Information

## Figures and Tables

**Figure 1:**
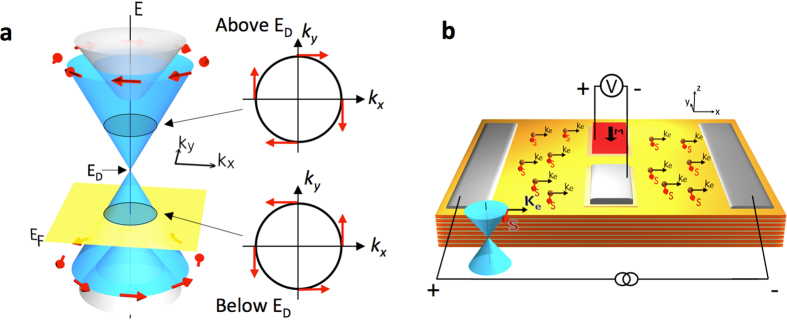
Schematic of the helical spin texture of TI surface bands and experimental concept. (**a**) Schematic diagram of the spin texture in TI, showing the chirality of the helical spin texture is inverted above and below the Dirac point. (**b**) Concept drawing of the transport experiment. The voltage measured at the ferromagnetic detector is proportional to the projection of the current-induced TI spin polarization onto the contact magnetization.
