# Supplementary material for: Electrical Detection of the Helical Spin Texture in a p-type Topological Insulator Sb2Te3
Source: Sci Rep. 2016 Jul 11;6:29533. doi: 10.1038/srep29533 (PMC4941728; doi:10.1038/srep29533)
Supplement: Supplementary Information [file srep29533-s1.pdf]

Supplementary Information

Electrical Detection of the Helical Spin Texture in a p-type Topological Insulator Sb_2Te_3

by C.H. Li, O.M.J. van ‘t Erve, Y.Y. Li, L. Li and B.T. Jonker

Conductivity of the 4C-SiC substrate

Conductive n-type 4C-SiC substrate is used to enable scanning tunneling microscopy imaging and tunneling spectroscopy to monitor in situ the surface morphology and electronic properties of the film used for transport studies. The conductivity of the 4C-SiC substrate however, does not contribute to the overall conductance of the p-type TI film in the transport geometry employed here, due to the depletion (or neutral) region at the p-n junction interface [1], as shown in the diagram below (Figure SI-1a). Note here since epitaxial graphene on SiC is doped n-type, as confirmed by angle resolved photoemission and tunneling spectroscopy experiments [2-5], it is hence considered together with the SiC as the “n-type substrate”.

To confirm this experimentally, we have conducted temperature dependent four point measurements (Figure SI-1b) in the same device as that in the main text, to demonstrate that the conductivity of the substrate does not contribute to transport. First we measure the resistivity of the n-type 4C-SiC substrate as a function of temperature, as shown in Fig. SI-1c. The resistivity shows weak temperature dependence, increasing from 0.028 Ω-cm at 300 K to 0.047 Ω-cm at 15 K, a change of less than a factor of 2.

From this resistivity we calculated the resistance of the substrate in the device where the 4pt measurement is conducted, shown as blue dots in Figure SI-1d. The overall 4pt resistance of the device (sum of all conduction path) is shown in green dots. Clearly the total 4pt resistance is ~ 3 orders of magnitude greater than that of the substrate, indicating that the substrate does not contribute to conduction. In addition, the temperature dependence of the resistance is also qualitatively different: a more than 2 orders of magnitude increasing in resistivity is observed from 300 to 15 K, a semiconducting behavior, whereas the substrate resistance only increase by a factor of less than 2. These results show that the n-type 4C-SiC substrate does not contribute to lateral transport through the p-type Sb_2Te_3 thin film.

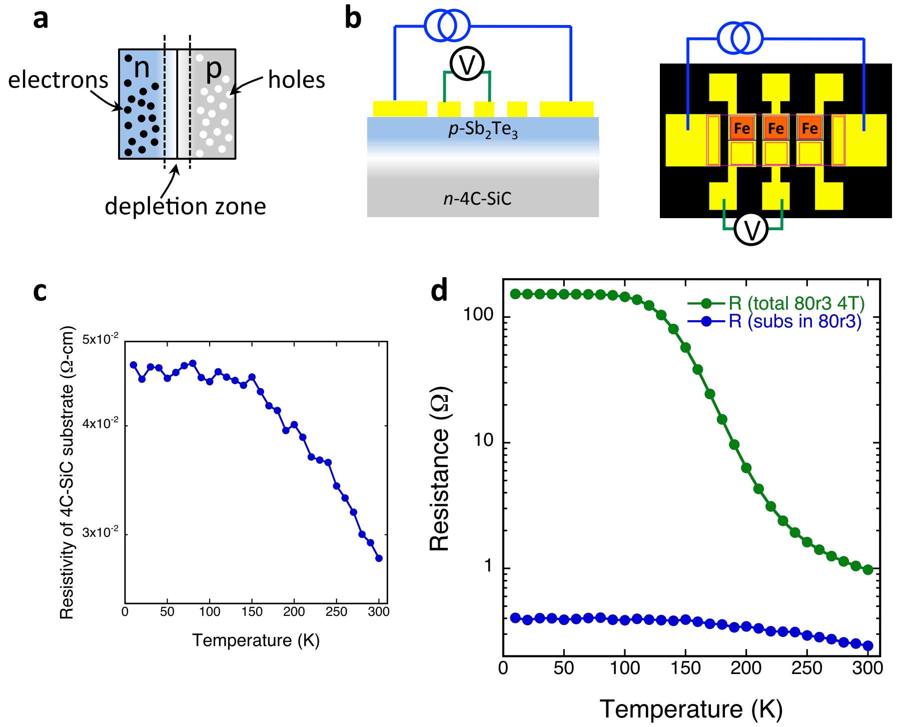

Figure S1. a) Schematic of depletion zone at p-n junction interface. b) Schematic (side and top view) of 4-point resistivity measurement. c) Temperature dependence of resistivity of the 4C-SiC substrate. d) 4-point (4T) resistance of the film/device (green dots) compared to that of the substrate (blue dots).

Hall measurements

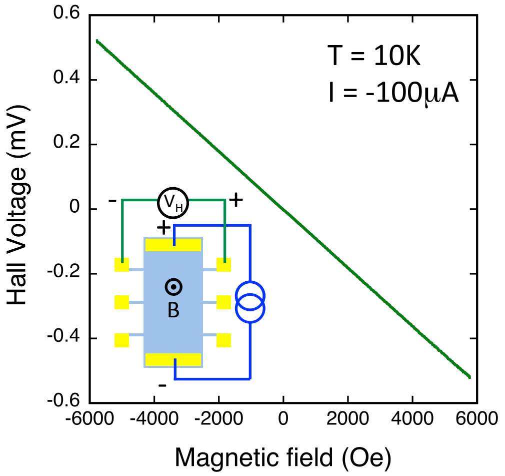

Figure S2. Hall measurement of the Sb_2Te_3 sample at 10K. The slope indicates hole conduction.

REFERENCES

1 Sze, S. M., Physics of Semiconductor Devices, 2nd Ed., John Wiley & Sons, 1981.

2 Ohta, T., Bostwick, A., McChesney, J. L., Seyller, T., Horn, K., Rotenberg, E., Interlayer interaction and electronic screening in multilayer graphene investigated with angle-resolved photoemission spectroscopy. *Phy. Rev. Lett.* **98**, 206802 (2007).

3 Lauffer, P. et al. Atomic and electronic structure of few-layer graphene on SiC(0001) studied with scanning tunneling microscopy and spectroscopy. *Phys. Rev. B* **77**, 155426 (2008).

4 S. Rajput, M. Chen, Y. Liu, Y. Y. Li, M. Weinert, and L. Li, Spatial fluctuations in barrier height at the graphene-silicon carbide Schottky contact, *Nat. Commun.* 4, 2752 (2013).

5 Li, Y. Y., Chen, M. X., Weinert, M., Li, L., Direct experimental determination of onset of electron–electron interactions in gap opening of zigzag graphene nanoribbons. *Nat. Commun.* **5**, 4311 (2014).
